# Pollination niche availability facilitates colonization of *Guettarda speciosa* with heteromorphic self-incompatibility on oceanic islands

**DOI:** 10.1038/s41598-018-32143-5

**Published:** 2018-09-13

**Authors:** Yuanqing Xu, Zhonglai Luo, Shaoxiong Gao, Dianxiang Zhang

**Affiliations:** 1 Key Laboratory of Plant Resources Conservation and Sustainable Utilization, South China Botanical Garden, The Chinese Academy of Sciences, Guangzhou, 510650 China; 2 Chongqing Nanshan Botanical Garden, Chongqing, 400065 China

## Abstract

Obligate out-breeding plants are considered relatively disadvantageous comparing with self-breeding plants when colonizing oceanic islets following long-distance dispersal owing to mate and pollinator limitation. The rarity of heterostyly, a typical out-breeding system, on oceanic islands seems a good proof. However, a heterostylous plant, *Guettarda speciosa*, is widely distributed on most tropical oceanic islets. Our research demonstrates that its heteromorphic self-incompatibility, plus herkogamy and long flower tube make it rely on pollinator for sexual reproduction, which is generally considered “disadvantageous” for island colonization. We hypothesize that available pollination niche will be a key factor for its colonization on islands. Our studies on remote coral islands show that *G*. *speciosa* has built equilibrium population with a 1:1 morph ratio. It could obtain pollination niche from the hawkmoth *Agrius convolvuli*. A pioneer island plant *Ipomoea pes-caprae* sustain the pollination niche by providing trophic resource for the larvae of the pollinator. Geographic pattern drawn by Ecological Niche Modelling further indicates the interaction between *G*. *speciosa*, *A*. *convolvuli* and *I*. *pes-caprae* can be bounded on those remote oceanic islands, explaining the colonization of *G*. *speciosa* distylous population. These findings demonstrated obligate out-breeding system could be maintained to acclimatize long distance dispersal, if the pollination niche is available.

## Introduction

Islands, especially oceanic islands, are considered as “natural evolutionary laboratories” by biologists due to their uniqueness such as isolated biotas, distinctive environments, and fluctuant communities^1,2^. How organisms colonize on islands following long-distance dispersal and adapt insular environments is a significant question for island evolutionary ecology. For plant colonizers, given that long-distance dispersal is generally by chance, Baker^3^ proposed that self-compatible, especially self-pollinated, hermaphrodite species have an advantage when colonizing and establishing in new habitats because one propagule is enough for successful sexual reproduction and establishment. This statement is now widely accepted as “Baker’s Law”^4^. In addition, with the paucity of pollinator on oceanic islands, especially those that are remote and small, plants capable of uniparental reproduction via generalist pollinators are believed to be more successful (reviewed by Pannell and Barrett^5,6^). It’s reported that the probability of self-compatible plants occurring on an island was more than twice that of self-incompatible species^7^. On the contrary, it’s more difficult for plants with self-incompatibility systems (i.e. obligate out-breeding system) to adapt such condition which require more than one individual for population establishment and sufficient pollination service for sexual reproduction^1,2,8–10^.

However, for those obligate out-breeding plants colonized oceanic islands successfully following long-distance dispersal, why they can adapt this process remain underexplored^6^. During their colonization, single individuals will lose mating opportunities unless that individual is able to await the later arrival of compatible mates. Thus, long life history (perennial vs. annual), high number of propagules and good dispersal abilities are essential to sustain the abundance and occupancy rate during initial colonization^6^. After first landing, sexual reproduction is the precondition to build stable population apart from the species with strong vegetative reproduction ability. Except few anemophilous species, most out-breeding plants rely on pollinator for sexual reproduction. Actually, mate limitation is primarily conducted by pollinator^7^. Even though the plant has established its initial group, the paucity of effective pollinator strongly limited pollen flow resulting in pollen limitation, in another word, mate limitation. Usually, plants with strong dispersal ability is considered to be more adapted to generalized pollinator in order to attain enough pollination service^6^.

The pollination resource that a plant can get from its effective pollinators reflects its pollination niche in community, consisting of fundamental niches and other physical environmental factors^11–15^. Like the general ecological niches, it represent the fit of species to natural selection, determining where species occur and whether they coexist^16,17^. The local pollinator community operates as a habitat filter on plant invasion and colonization accomplished with certain characters of breeding system, and then pollinator-mediated interactions will impact on species establishment and character persistence^13,18,19^. Obviously, for plants that are self-incompatible and hence outbreeding, pollination niche availability is a necessary condition to establish in new habitats through sexual reproduction. Obviously, it also will be especially important for obligate out-breeding plants to establish on oceanic islands after long-distance dispersal. It’s reasonable to hypothesize that their adaptation modes are related to the availability of pollination niche^20,21^.

In order to explore the hypothesis mentioned above, a heterostylous plant *Guettarda speciosa* L. is selected as a model in this study. The first reason why we choose this plant species is that heterostyly system, a genetically controlled floral polymorphism, is considered as a typical mechanism for promoting out-breeding^22,23^. Populations of heterostylous species are composed of two (distyly) or three (tristyly) distinct floral morphs that differ reciprocally in the heights of stigmas and anthers with a significant herkogamy in flowers. Generally, most heterostylous species are obligate out-breeding due to heteromorphic (self and intra-morph) incompatibility^24–26^, posing the intrinsic barrier for its persistence on small oceanic islands^27–29^. Even for self-compatible species, pollinator paucity will generally lead to mate limitation in sexual reproduction. As the flowers of heterostylous plants are often tubular and herkogamy, proper pollinators with matched proboscis length are required for both morphs of heterostylous plant to be successfully pollinated^30,31^. Isoplethic morph-ratio is another prerequisite for the maintenance of heterostylous populations^32,33^; in other words, few compatible individuals with unbalanced morph ratio will also lead to population depression. All these characteristics added constraints for heterostylous plants to acclimatize on small oceanic islands through sexual reproduction. Indeed, heterostylous plants are rarely recorded on islands^29,34^. Besides, heterostylous system on islands broke-down with lost or weakened self-incompatibility and heteromorphism in some cases, which is regarded as a kind of adaptive change to overcome the unfavorable conditions on oceanic islands such as paucity of proper pollinators^28,35,36^. The second reason is that *G*. *speciosa* is a widely distributed island plant^29^. Despite that it’s long tubular flower lead to a dependence on pollinator for its sexual reproduction (see Results), it distributes on almost all tropical oceanic islands, and even becomes a dominant species, suggesting its successful adaptation modes for long distance dispersal and island habitat. The heterosylous system of *G*. *speciosa* provides an ideal model to test our pollination niche hypothesis.

Similar to the other basic ecological niche, pollination niche sustains the plants, and in turn restricts the plants’ distribution, which means the geographic distribution of the plants will be interfered by the pollinators likewise climate^12,13^. Similar interspecies relationship often exists between the host and the parasites. The host is considered as a key environmental factor to predict the potential distribution of parasites in many studies^15^. Analogously, if pollination niche availability is essential for the acclimatization of heterostylous plants on islands, their relationship and interaction is expected to be interpreted by geographic distribution patterns. Here we apply Ecological Niche Modeling (ENM) to explore the test. ENM is a multidisciplinary tool mainly applied to predict species’ geographic distributions or niche space, offering reliable global scale information for biogeographical, evolutionary, and ecological analyses^37–39^. The pattern of biotic interactions, such as host-parasite, flower-pollinator, co-occurring, in geographic scale can be investigated by ENM^40–43^, though precise quantification is considered as unlikely by current methodology^44^. We expect that with the effect of pollination niche, the obligate out-breeding plant will concentrated in the pollination niche available areas.

In the meanwhile, pollination niche availability on small oceanic islands was confined by many factors. Besides the pollinators’ dispersal ability and basic climatic conditions on islands, food resource is a key limiting factor, especially for insects with specific hosts, such as most Lepidoptera pollinators. The importance of food plants as range determinants has been illustrated by researches on butterflies and the host plants of their larvae^45,46^. The trophic dependence of their larvae encourages the insects and plants develop close evolutionary relationships (e.g. co-evolution, co-existence)^47–49^. And therefore it’s necessary to take the pollinator’s host plants into consideration when analyzing the geographic signature of such kinds of pollinators.

In this study, we explored why *G*. *speciosa* can colonize widely and successfully on oceanic islets following long-distance dispersal with heterostyly system. Its floral traits, morph ratio, compatibility system and pollinators (hawk moths) were investigated, and the host plants of pollinators was surveyed. Ecological Niche Modelling was applied to predict the distribution of *G*. *speciosa*, its pollinators and the host plants of pollinators, testing if its success relates to pollination niche availability. We expect that if pollination niche affects its colonization on oceanic islets: (i) The distribution range of *G*. *speciosa* will be contained in the pollinators’ distribution (nested models) and its occurrence will concentrate in pollinator-available regions due to its dependence on pollinator; (ii) if there’s any host plant that is necessary to sustain the pollination niche, a co-existence pattern is expected between *G*. *speciosa* and the pollinators’ host plants.

## Results

### Floral traits and flower longevity

**Figure 1:**
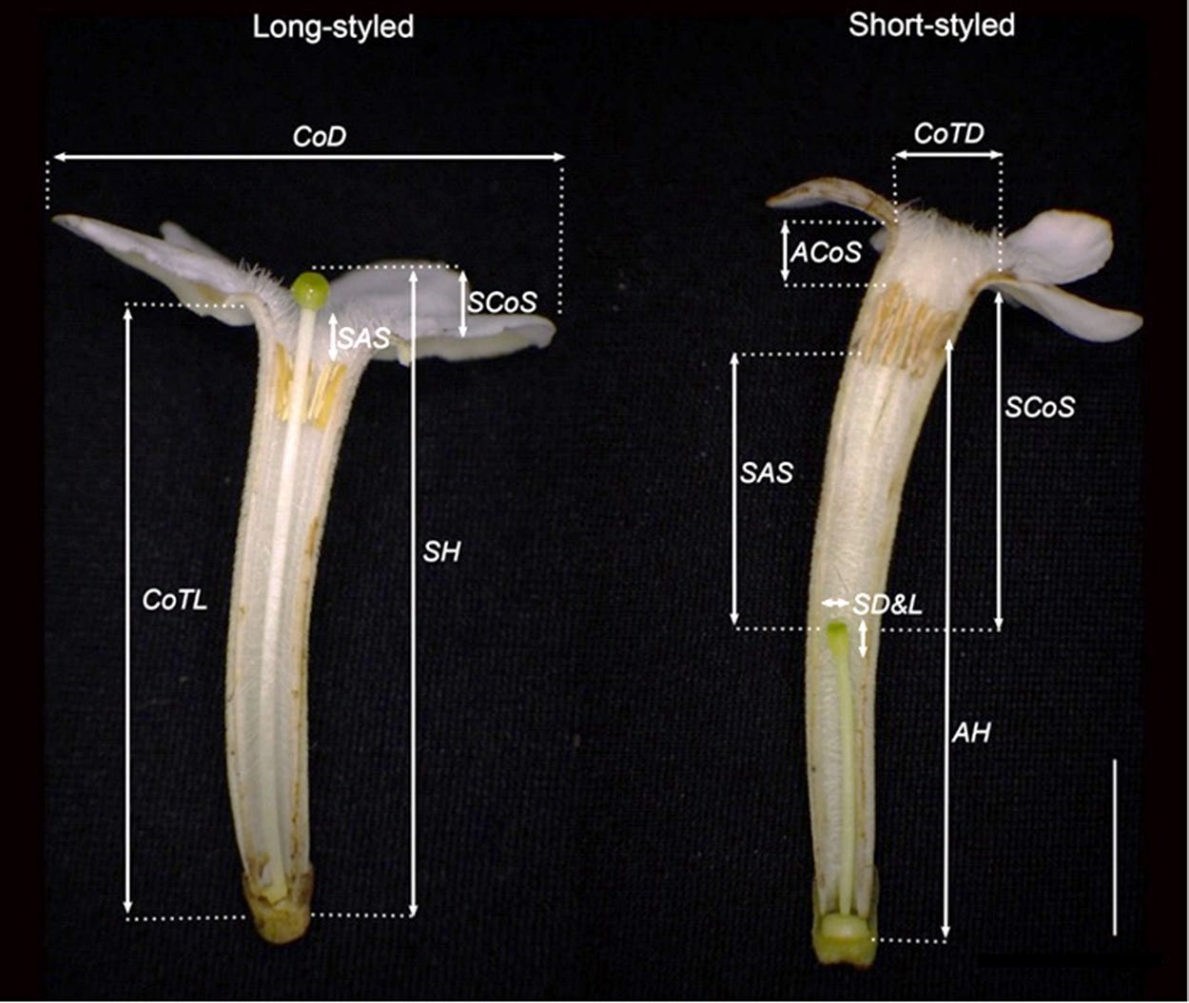
Floral morphology of *Guettarda speciosa*, showing the position of anther and stigma, and measurements of floral characters. CoD: corolla diameter; CoTL: corolla tube length; CoTD: corolla tube diameter; SCoS: stigma-corolla separation; SAS: stigma-anther separation; AH: anther height; SH: stigma height; CoAS: corolla-anther separation; SD&L: stigma’s diameter and length. Bar = 10 mm.

The *G*. *speciosa* populations in Xisha Islands involved two discrete floral morphs differing in style length: L-morph flowers have stigma positioned above the anthers and slightly exserted out of corolla, while S-morph flowers have stigma positioned below the anthers. However, anthers of both morphs are arranged on the upper part of the corolla tube near the mouth but are not exserted (Fig. 1, see M & M for detail). The Reciprocity Indices (v2.0) recorded were: for the whole level R = 0.521, the higher level R = 0.845 and the lower level R = 0.697.

The ancillary polymorphism occurs in almost every part of the flowers, including the corolla size, anther length, stigma and pollen size [Supplementary Data, Table S1]. The S-morph presents larger flower, longer anther and stigma than the L-morph. However, the exine sculpture of pollen and the papilla of stigma show similar appearance between the two morphs [Supplementary Data, Fig. S1].

In the study sites, white flowers of *G*. *speciosa* opened around 19:00-20:00 in the night with strong aroma and anther dehisced. Sticky pollen grains adhered together to form bars in anthers. Stigma is also sticky when flower opened. Secretion was observed on top of the anthers [Supplementary Data, Fig. S2]. Nectar was secreted in the bottom of corolla tube. Corolla withered around 09:00 next morning but the styles persisted. Legitimate pollen grains scarcely germinated on the stigmas of flowers which had opened for 12 hours, suggesting that the pollen viability or stigma receptivity only persisted during the first night. Flower longevity was thus determined to be less than 12 hours.

### Morph ratios

In the three island populations we investigated, the ratios of L-morph and S-morph individuals did not deviate from the expected 1:1 equilibrium (Table 1). The results indicate that symmetrical disassortative mating occurs in the three populations.

**Table 1:** Ratios of L-morph and S-morph individuals on the Yongxing, Ganquan and Jinqing Islands.

	Yongxing (n = 107)	Ganquan (n = 83)	Jinqing (n = 55)
S-morph	60	38	30
L-morph	44	45	25
S:L ratio	1:0.75	1:0.84	1:0.83
*G* test*	χ^2^ = 2.462, P = 0.117	χ^2^ = 0.5904, P = 0.442	χ^2^ = 0.4546, P = 0.500

^*^Not significant on the 0.05 level.

### Heteromorphic self-incompatibility

The results of hand pollination indicated that *G*. *speciosa* in Xisha Islands exhibits heteromorphic incompatibility. Both morphs are self-incompatible and none of the intra-morph pollinated (illegitimate cross) flowers set fruit. Under inter-morph pollination treatments, 85.71% of L-morph flowers and 89.66% of S-morph flowers set fruits (Table 2). Significantly, in both floral morphs fruit set of open-pollinated flowers was very low, suggesting pollen limitation in *G*. *speciosa*.

**Table 2:** Fruit sets of *Guettarda speciosa* with different pollination treatments.

Treatment	Fruit sets (%)	
	L-morph	S-morph
Self-pollination	0 (n = 35)	0 (n = 23)
Intramorph pollination	0 (n = 32)	0 (n = 27)
Intermorph pollination	85.71 (n = 28)	89.66 (n = 29)
Net	0 (n = 26)	0 (n = 27)
Natural Control	18.58 (n = 210)	9.67 (n = 189)

**Figure 2:**
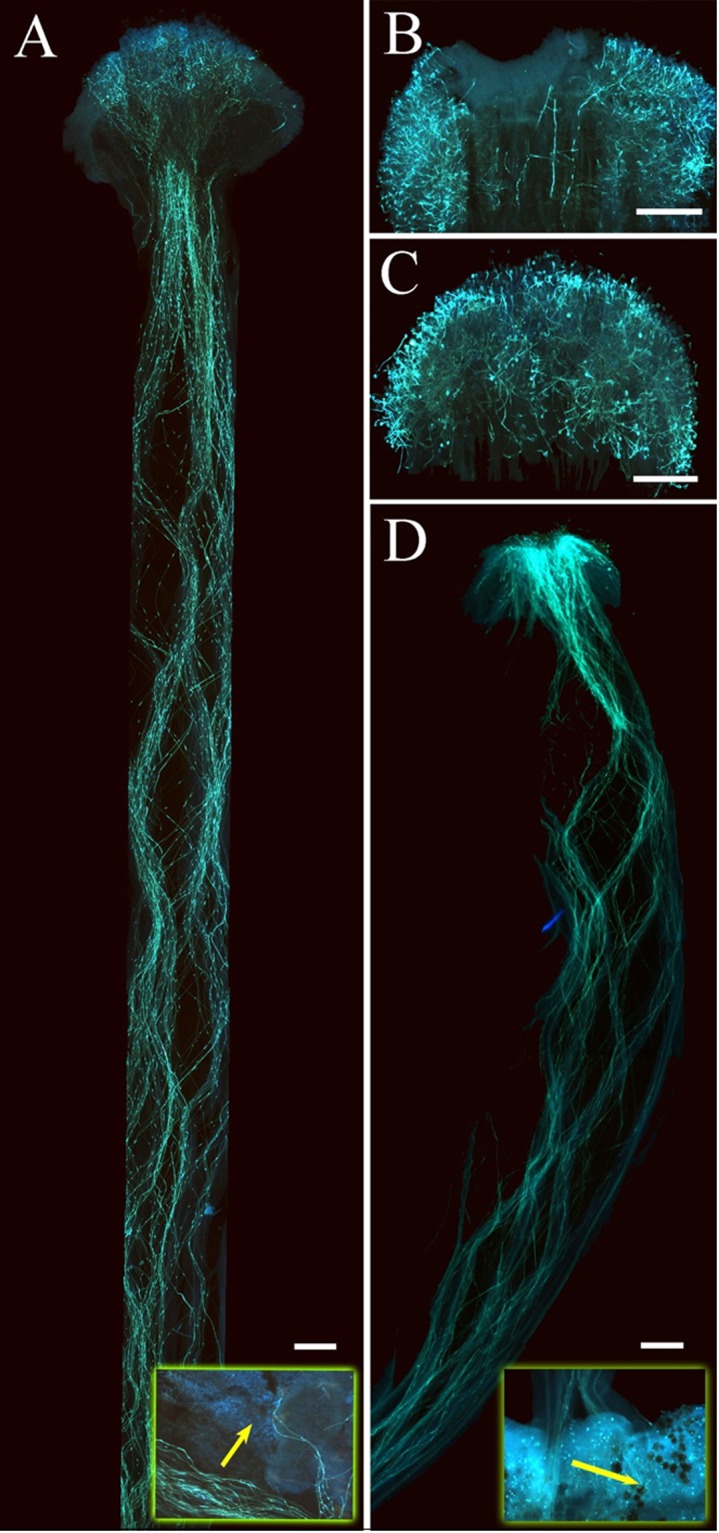
*Guettarda speciosa* pollen tube growth in *vivo* after hand pollination. (**A**) L-morph style 24 h after inter-morph pollination; (**B**) L-morph style 24 h after self-pollination; (**C**) S-morph style 24 h after self-pollination; (**D**) S-morph style 24 h after inter-morph pollination. Bar = 1 mm.

Results of artificial pollination experiments with time intervals suggest that pollen tubes were inhibited in the stigma after self- and intra-morph pollination in the first hour. In contrast, after inter-morph pollination, pollen grains germinated within 1 h and 24 h later pollen tubes entered the ovary (Fig. 2).

### Pollinators and their host plants

**Figure 3:**
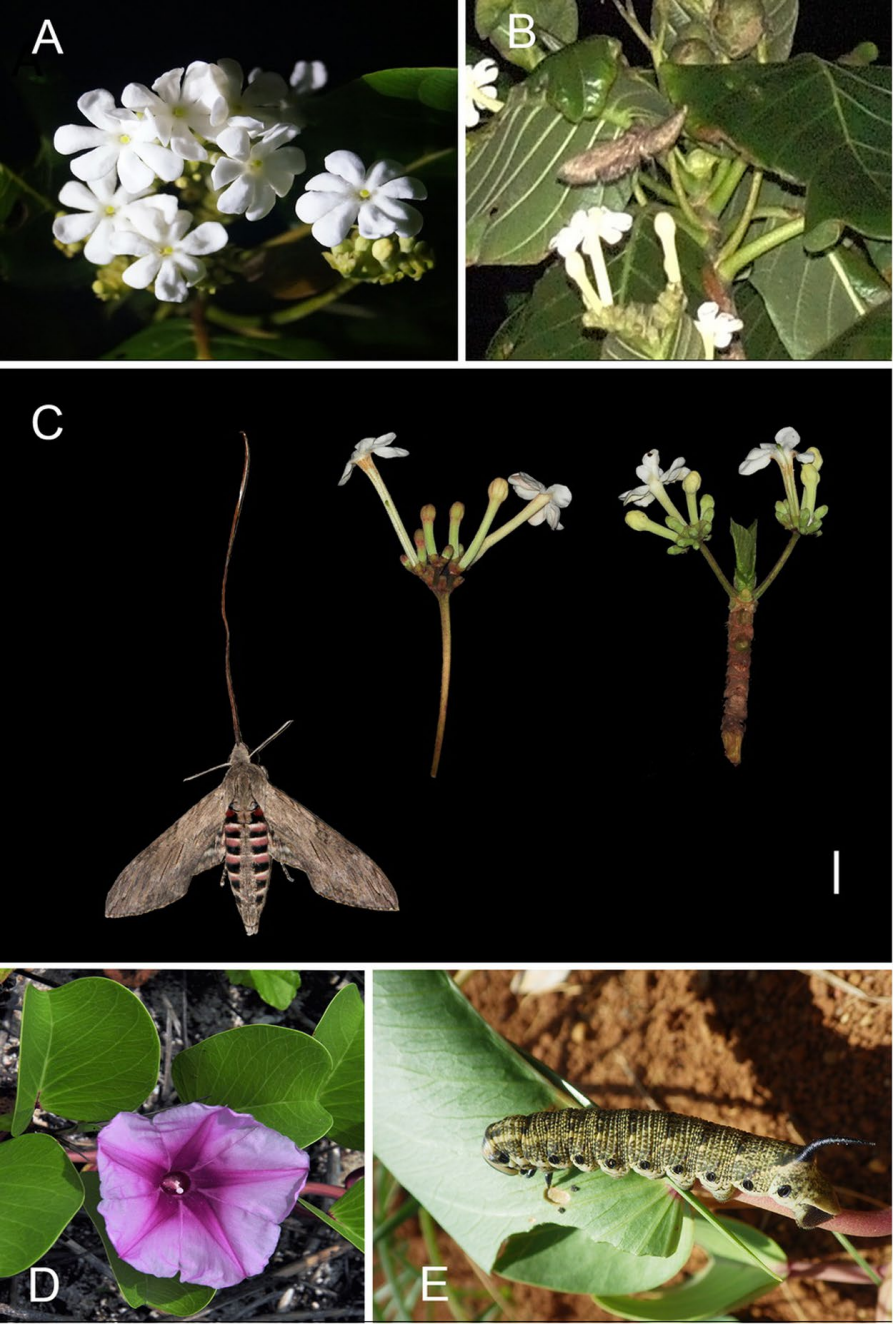
The flower and pollinator of *Guettarda speciosa* in study site. (**A**) Blooming inflorescence shows narrow corolla throat; (**B**) *Agrius convolvuli* is pollinating the flowers; (**C**) length comparison of the tongue of *A*. *convolvuli* and corolla tube of *G*. *speciosa*. Bar = 10 mm. (**D**) *Ipomoea pes-caprae* in natural habitat; (**E**) larva of *A*. *convolvuli* feeding on the leaves of *I*. *pes-caprae*, the host plant.

Visitation frequencies of pollinators were very low. Our observation covered the three largest populations of *G*. *speciosa*, with around 60 flowers in anthesis each night within our observation area (Fig. 3A). In 14-night observations, we witnessed hawkmoth *Agrius convolvuli* L. at only two nights around 22:00 (Fig. 3B,C). *Agrius convolvuli* visited nearly all the open flowers in each visitation, flying swiftly among inflorescences. They feed from the flowers with their long tongues inserting deeply into the corolla tube, making contact with the anthers and stigma. Visits typically lasted several seconds for each flower, and the open flowers were visited sequentially. As the longevity of single flower is only one night, we define the visitation frequencies as: V = No. of days that *A*. *convolvuli* presented/Total No. of observation days, which is 0.14.

In 7d observations at dawn, various kinds of visitors were witnessed, including bees and flies, but they just acted as robbers for nectar or pollen, as none of them could touch the sexual organs due to their small body size. Hawkmoth *Cephonodes hylas* L. is a visitor touched the sexual organs, but we just witnessed it visiting the flowers at around 08:00-10:00, while the flower had lost viability. Thus, it denies the *C*. *hyla* as an effective pollinator. We surveyed every recorded plant species (about 220) on Xisha Islands, and found the larvae of *A*. *convolvuli* parasitized only on the leaves of *Ipomoea pes-caprae* L. (Convolvulaceae) (Fig. 3D,E).

### Distribution pattern of *G*. *speciosa*, the pollinator and its host

**Figure 4:**
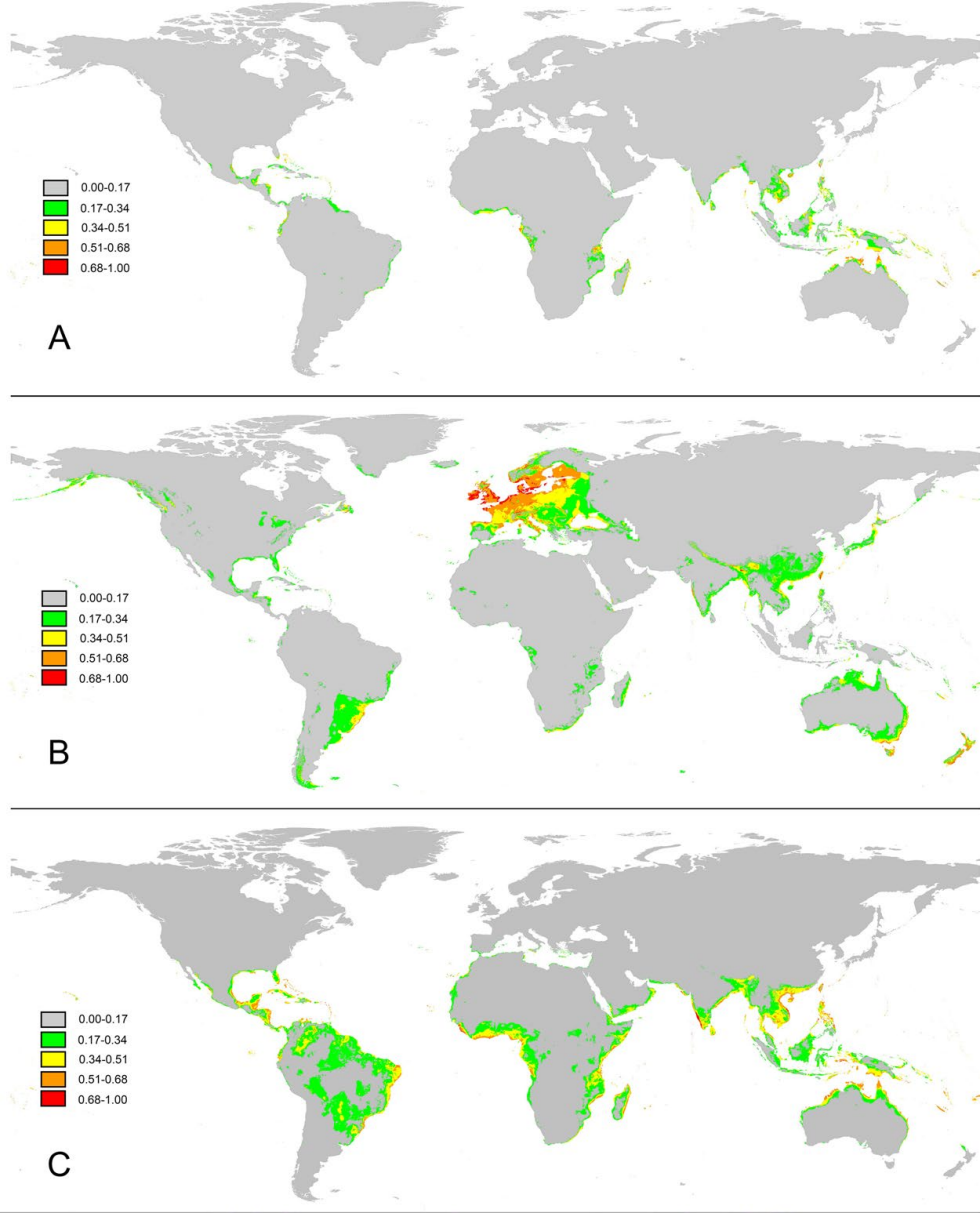
ENM predicted distribution regions of *Guettarda speciosa* (**A**), *Agrius convolvuli* (**B**) and *Ipomoea pes-caprae* (**C**).

The modelled potential distribution of the three species is shown in Fig. 4. In total, 988 records for *G*. *speciosa*, 15208 records for *A*. *convolvuli* and 5339 records for *I*. *pes-caprae* were used in the modelling. Model evaluation showed high scores of performance, and all the AUC values were above 0.9. In the predicted distribution, *G*. *speciosa* is in most Pacific islands, some tropical coast areas of Australia, India, Africa and some islands in the Caribbean Sea. The hawkmoth pollinator *A*. *convolvuli* has a world-wide range and spreads almost everywhere in Western Europe, with distribution in sub-tropical and tropical areas from Southeastern Asia to Eastern Australia, Southern Africa to Eastern Madagascar, and Southeastern coast of North America. The host of *A*. *convolvuli*, *I*. *pes-caprae*, distributes in similar but larger areas with *G*. *speciosa*. ENM predicts the three species have the ability to dispread on many ocean islands.

**Figure 5:**
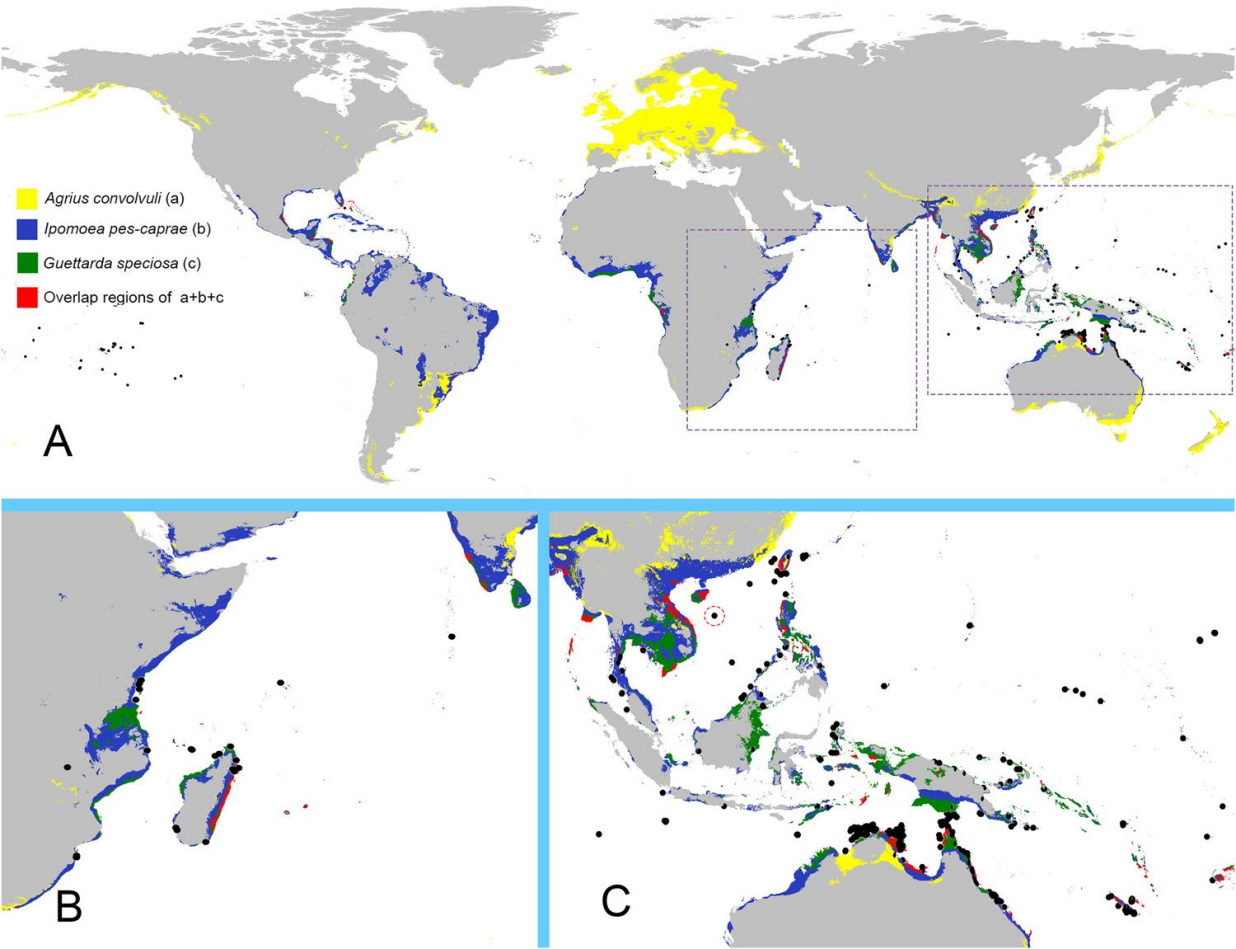
ENM predicted distribution regions for *Guettarda speciosa* (green), *Agrius convolvuli* (yellow) and *Ipomoea pes-caprae* (blue) in 10th percentile training presence. Red color highlights the overlapping areas of the three species, and black dots indicate the occurrence records of *G*. *speciosa*. Figures B and C illustrate the details of the regions in the dashed boxes in figure A. Red dashed circle in figure C highlights the experimental site, Yongxing Island.

Figure 5 shows the ENM predicted distribution regions of the three species in 10^th^ percentile training presence. Overlapping areas with *G*. *speciosa* is highlighted in red and the occurrence records of *G*. *speciosa* are marked by black dots. The ratio of overlapping area to predicted distribution area of *G*. *speciosa* for different species was 91.84% (*I*. *pes-caprae* vs. *G*. *speciosa*), 22.45% (*A*. *convolvuli* vs. *G*. *speciosa*), 22.40% (*I*. *pes-caprae* and *A*. *convolvuli* vs. *G*. *speciosa*), respectively.

## Discussion

Our results indicate that *G*. *speciosa* is distylous with self- and intra-morph incompatibility, and the heterostylous traits are stable on oceanic islets. *Guettarda speciosa* is a nocturnal flowering plant with hawkmoth *A*. *convolvuli* as the principal legitimate pollinator in the study area. ENM results show the recorded occurrence of *G*. *speciosa* on oceanic islands mostly locates in the predicted distribution area of *A*. *convolvuli*. What’s more, it reveals a geographic signal that relationships between *G*. *speciosa*, *A*. *convolvuli* and *I*. *pes-caprae* observed on Yongxing Island is able to be bound on other insular regions, suggesting the persistence of obligate out-breeding system in *G*. *speciosa* on oceanic islands colonization is closely related to the available pollination niche.

### *G*. *speciosa* is an obligate out-breeding species with stable distylous population on islands

Floral traits indicate that *G*. *speciosa* is morphologically distylous, with imprecise reciprocal herkogamy. The stigma-anther reciprocity is much more precise in higher level (R = 0.845) than in lower level (R = 0.697). Comparing to typical distyly, the distyly in *G*. *speciosa* is a kind of “anomalous” heterostyly, a phenomenon designated by Barrett and Richards^50^ for species displaying imprecise reciprocity. Resembling many typical distylous species, *G*. *speciosa* also showed dimorphism in corolla size, anther length, as well as stigma and pollen sizes.

*Guettarda speciosa* is strictly self- and intra-morph incompatible, and the equilibrium of morph ratio in the three populations suggests that the species is capable of maintaining stability on Xisha Islands. It is widely distributed in coastal habitats in tropical areas around the Pacific Ocean. Although reproductive biological data of *G*. *speciosa* is limited on other islands, the report of distyly on Lanyu Island^28^ suggested that distyly in *G*. *speciosa* is persistent in different populations. Besides, Yongxing Island and other small and young islets we studied here are all far from the continent. The dominance of self-incompatible *G*. *speciosa* on remote islets suggests that these reproductive characters are entrenched after long distance dispersal.

Two congeneric species, *G*. *scabra* and *G*. *platypoda*, are also coastal and insular woody plants with distyly^51,52^. Similar “anomalous” distyly has been reported in these species as well, indicating the possibility that the imprecise reciprocity is a general feature of this genus. *Guettarda scabra* and *G*. *platypoda* are self- and intra-morph compatible, and capable of autonomous selfing. Both species, however, are relatively stenochoric, compared to the self-incompatible *G*. *speciosa* which is more widely distributed on oceanic islands as a dominant plant^53–55^, suggesting its advantage to acclimatize long distance dispersal under marine environments. This is in contrast with the findings of Grossenbacher *et al*.^56^, which reported that plants autonomously reproduced via self-pollination consistently had larger geographic ranges than their close relatives which generally required two parents for reproduction. However, it should be noted that fluctuation of pollinator service drives out-crossers to increase fitness via dispersal, so out-crossers show a stronger dispersal ability than selfers^20^. The floral traits, especially long flower tube, of *G*. *speciosa* reduce its fitness to general pollinator group, increasing the risk of pollination fluctuation. This may drive *G*. *speciosa* to possess a stronger dispersal ability than its relatives and achieve a wider distribution range.

### The nocturnal flowering *G*. *speciosa* obtains pollination niche from hawkmoth *A*. *convolvuli*

The tubular flower of *G*. *speciosa* opened around 19:00–20:00 in the night, with white color and strong aroma, showing typical hawk moth-pollination syndrome^57,58^. The narrow floral tube, unexposed anthers, as well as the deep-hidden nectar, reinforced its dependence on hawkmoth pollinators. Though both *A*. *convolvuli* and *C*. *hyla* were observed visiting *G*. *speciosa*, our data demonstrated that only the nocturnal *A*. *convolvuli* is the legitimate pollinator, while *C*. *hyla* is not an effective pollinator, as the stigma has lost receptivity in daytime when *C*. *hyla* visited the flowers. *Agrius convolvuli* visited *G*. *speciosa* at a very low frequency in this study, which may reflected the restriction of islands on large insects^59^, or the discrepant pollination syndrome of *G*. *speciosa* and *A*. *convolvuli*. *A*. *convolvuli* is a large hawkmoth with very long tongue (approx. 10 cm) and very strong dispersal ability from temperate to tropical zone. It acts as the principal pollinator for various angiosperm groups with long floral tube, including *Crinum delagoanse*, *Gardenia thunbergii*, *Ipomoea alba*^18^, *Bonatea steudneri*, *Datura stramonium*^18^ and *Lilium formosanum*^60^. The floral tube length of *G*. *speciosa*, however, is shorter, suggesting it adjusted to pollinators with relatively shorter tongue (minimum 4 cm); and thus doesn’t well match the tongue length of *A*. *convolvuli*.

However, clear specialization tendency was observed on Yongxing Island that *A*. *convolvuli* was the only effective pollinator for *G*. *speciosa*. This may be explained by the rarity of long tubular flowers (10 cm) (only two species^61^) on the islands that *G*. *speciosa* played as important trophic resource for hawkmoth. For *G*. *speciosa*, *A*. *convolvuli* is an effective pollinator. Even though it was an irregular visitor, it visited nearly all the flowers during each visitation bout in the observation area. The sticky pollen and stigma of *G*. *speciosa* further increased pollination efficiency. The observed visitation frequency (0.14) is quite match with the low natural fruit set (0.14 for two morphs on average), which indicated that *G*. *speciosa* successfully and primarily obtains the pollination niche from *A*. *convolvuli*. Besides, *G*. *speciosa* blooms nearly all year round with abundant flowers opening every day, further ensuring enough offspring for the maintenance of distyly.

### The geographic signal of the relationship between *G*. *speciosa*, *A*. *convolvuli* and *I*. *pes-caprae*

The ENM results show that *A*. *convolvuli* have much broader potential distribution areas than that of *G*. *speciosa*. From an overall perspective, the potential distribution of *G*. *speciosa* isn’t completely contained within the predicted range of the pollinator, *A*. *convolvuli*, showing a mosaic model. Species with simple and tight interactions showing similar distributive preference usually exhibit nested patterns^41,62^, while the mosaic model is on the contrary. Our results suggest that relationship between *G*. *speciosa* and *A*. *convolvuli* is not a strictly specialized relationship in global scale, which is coherent with that in different area, *G*. *speciosa* may be pollinated by other potential insects, and *vice versa*, *A*. *convolvuli* may visit other plants in other habits.

However, the predicted distribution of *G*. *speciosa* is better overlapped with that of its pollinator on small and remote oceanic archipelagos than mainland and large islands (i.e. New Zealand, Papua New Guinea, etc.) (Fig. 5). This suggests a more specialized interaction between *G*. *speciosa* and *A*. *convolvuli* can build on islands, as observed in our study sites. The specialization degree in pollinator-plant relationship is variable in different biogeographic ranges^9,63^. Because the limited species number and lower animal/plant ratio on oceanic islands, the pollinator-plant interactions were simpler with lower diversity, though there were more generalized pollinator species than mainland and continental islands^63,64^. Therefore, a specialization-like relationship between pollinator and plant will be observed, as plant has no choise but depend on fewer pollinators.

**Figure 6:**
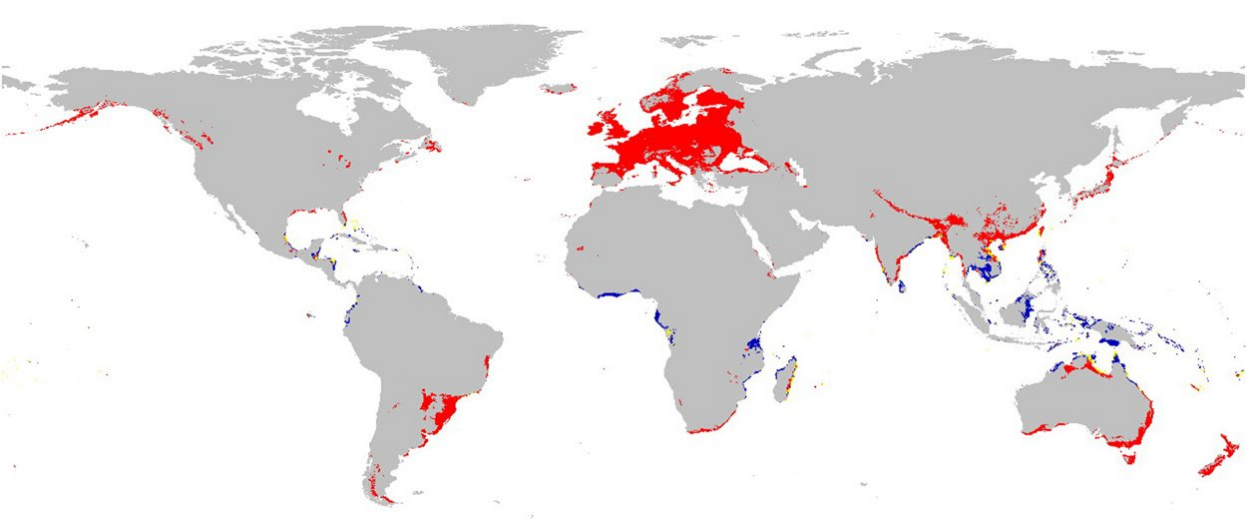
Suitable range for *Agrius convolvuli* (Red), *Guettarda speciosa* (Blue), overlapping regions (Yellow) in 10^th^ percentile training presence.

Interestingly, the overlapping pattern doesn’t change after adding the distribution data of *Ipomoea pes-caprae*, the host of *A*. *convolvuli*, comparing to the pattern between *A*. *convolvuli* and *G*. *speciosa* (Fig. 6). Moreover, occurrence records of *G*. *speciosa* are concentrated in the overlapping areas of the three species. This condition indicates that *G*. *speciosa* is sympatric in areas where *A*. *convolvuli* overlaps with *I*. *pes-caprae*. *Ipomoea pes-caprae* has larger potential areas which covers 90% areas of *G*. *speciosa*, fitting well with the typical nested model. In many field investigation records and floras on oceanic islands, *I*. *pes-caprae* and *G*. *speciosa* have been reported to co-exist^54,55,65–67^. Our prediction on their distribution is in good accordance with the empirical data.

### The interactions of the focal plant, its pollinator and the host plant of the pollinator on oceanic islands

On Yongxing Island, *G*. *speciosa* obtains pollination service from *A*. *convolvuli*. On the larger scale, the geographic pattern indicates that the ternary relationship between *G*. *speciosa*, *A*. *convolvuli* and *I*. *pes-caprae* observed on Yongxing Island is able to be bounded on other insular regions, which is a reasonable explanation for the maintenance of distyly in *G*. *speciosa* as the species is able to obtain pollination niche from *A*. *convolvuli* on those remote oceanic islands. For the pollinator, *A*. *convolvuli*, trophic resource will be provided by the widespread *I*. *pes-caprae* on islands.

A question to consider is how such relationship between *G*. *speciosa*, *A*. *convolvuli* and *I*. *pes-caprae* developed. Is it developed as a result of interaction or a coincidence by chance? A result of interaction refers to a consequence in evolutionary history (e.g. co-evolution) and the dependence between species has played as a limitation on geographic distributions. A coincidence means that the plant, pollinator and host didn’t affect the distribution of each other and spontaneously develop association in their co-existence regions. In other words, the distribution of each species is mediated by its own autecology so that pollination and parasitism occurs on overlapping regions where climatic conditions are suitable for both of them^42^, indicating that the association builds from ecological fitting process^68^. Our present evidence may not be enough for creating a solid conclusion, as it is still difficult to quantify the interaction among the three partners^44^ though species interaction is considered as a factor influencing the geographic ranges^45^. However, it’s reasonable to postulate that the pollinator *A*. *convolvuli* would “mediate” the co-existence of *G*. *speciosa* and *I*. *pes-caprae*, besides their similar climatic preference. It’s reported that *I*. *pes-caprae* is a pioneer species on the community succession in oceanic islands^55^. After the establishment of *I*. *pes-capra*e population, they will facilitate the colonization of *A*. *convolvuli*. Then, the pollination niche for *G*. *speciosa* becomes available so that it can colonize the islets while maintaining its heterostylous self-incompatibility system. This scenario provides reasonable evolutionary explanation for the co-existence of *G*. *speciosa* and *I*. *pes-caprae* with *A*. *convolvuli* as key mediator. As plant-pollinator interactions will affect plant species establishment and persistence^19^, cross-regional population studies will shed more light on the inter-relationships among *G*. *speciosa*, *A*. *convolvuli* and *I*. *pes-caprae*.

## Conclusion

Baker’s law suggested that the capacity for self-fertilization would be favored but self-incompatibility would be filtered out in island floras where mates are scarce^6^. For heterostylous system, it’s considered disadvantageous and easily lost self-incompatibility for adaptation. Even dioecious species often display ‘leaky’ gender to keep self-fertilizing ability^69,70^. However, there’s no break-down of heterostyly or “leakiness” in the SI system of *G*. *speciosa*, demonstrating an “obligate out-breeding system”.

According to the co-occurrence pattern between *G*. *speciosa*, its pollinator and host plant of the pollinator in global scale, our present study shows that the obligate out-breeding system distyly in *G*. *speciosa* didn’t become a disadvantage and could be persistent to acclimatize long distance dispersal as *A*. *convolvuli* can provide pollination niche for *G*. *speciosa* in a wide scale. Compared to its self- compatible and autogamous sister species, *G*. *speciosa* shows much stronger dispersal ability and broader distribution range, which is a striking contrast to previous knowledge^1,8,56,60^. In this case, pollination niche availability seems to be a more important factor affecting on the plants distribution range rather than mating system. It provides an alternative comprehension for the natural selection on plant mating system during dispersal and expanding. Our study wouldn’t deny that an out-breeding system will add disadvantage on the new colonizers, but whether it will hinder plants’ dispersal and spread remains negotiable.

## Materials and Methods

### Study sites and species

Field studies were carried out from November 2014 through December 2015, and again on January and August 2017 at Yongxing Island (Woody Island) on Xisha Islands (Paracel Islands). Xisha islands are a series of coral islets, locating in South China Sea (15°40′–17°10′N, 110°–113°E). It was formed about 7000 years ago as the coral growth and crust uplift^71^. The plant species richness in Xisha Islands is very limited (about 220 species)^61^. While morph ratio investigation was conducted at Yongxing Island plus two nearby islets (Ganquan Island and Jinqing Island).The Yongxing Island (16°50′N, 112°20′E), 320 km from Hainan Island, the nearest mainland, with a total area of 1.9km^2^, is the largest islet of this archipelago.

*Guettarda speciosa* is a rubiaceous tree 2–6 m in height, with axillary cyme inflorescences. The fragrant white flower has a 3–4 cm long corolla tube and 8–10 corolla-lobes. Flowers open in the evening till next morning with a typical hawk moth pollination syndrome. Its sweet-smelling globular fruit is dispersed by animals and can stay afloat^54^. *G*. *speciosa* is widely distributed in the tropical islands and coastal zones around the Pacific Ocean, from the coastline of central and northern Queensland and Northern Territory in Australia, to Pacific Islands, including French Polynesia, Micronesia and Fiji, the Malesia, and the east coast of Africa. In Xisha Islands, it is one of the dominant species among the arborous layer^72^. Its style dimorphism has been reported previously by Watanabe and Sugawara^29^.

### Floral traits and flower longevity

To examine the floral variation in population we randomly selected 10 trees of each morph, and measured 3–5 flowers from different inflorescences for each plant. In total, 74 flowers of long-styled morph (L-morph) and 42 flowers of short-styled morph (S-morph) in anthesis were collected and 10 morphological traits (Fig. 1) were immediately measured by digital calipers (0.01 mm accuracy). We used the Reciprocity Index, calculated by Recipro-V2^73^, to represent the stigma-stamen reciprocal degree between the two morphs. Furthermore, some mature flower buds fixed in formalin/acetic acid/alcohol (FAA) solution were used to characterize auxiliary difference between morphs by scanning electron microscope (JSM-6360LV, Japan). For each morph, we mixed pollen grains of mature, intact anthers from five plants, and 30 pollen grains were measured. The stigma surface and pollen were observed and digital images were taken. Pollen equatorial axis and polar axis were measured in Image-Pro Plus (v. 6.0).

While the corolla tube persisted till the next morning, insect visits were witnessed in the morning. Therefore, flower longevity was checked by pollen-tube growth after inter-morph hand pollination to determine the effectiveness of diurnal pollinators. Freshly opened flowers (at 8 pm) and caged flowers 12 h after open (at 8am next morning) of L- and S-morphs were hand-pollinated by legitimate pollen grains. Five flowers for each treatment were picked from five individuals. Twelve hours after hand pollination, styles were harvested and then preserved in FAA. Observation on pollen-tube growth following the methods specified in the next section^74^.

All statistical analyses were performed using SPSS (version 13.0). Means (±SE) were calculated for all measurements. We compared the morphological differences between the two floral morphs using Mann-Whitney U test as most data aren’t normally distributed.

### Morph ratios

*Guettarda speciosa* populations in three islets of the Xisha Islands were investigated to determine the relative abundance of the two morphs. We randomly sampled the individuals in each population by walking through the whole habitat from east to west and from south to north. Yongxing Island (1.9 km^2^) population: n = 104 individuals, Ganquan Island (0.29 km^2^) population: n = 83 individuals, Jinqing Island (0.20 km^2^) population: n = 55 individuals. Morph ratio data were analyzed using the *G*-test for inequality of frequencies.

### Heteromorphic self- incompatibility system

From November 2014 to February 2015, hand pollination was performed on five labeled individuals of each morph in a natural population at the Yongxing Island. Forty inflorescences with unopened flowers were enclosed separately in 40-mesh bags. We performed four treatments: (1) self-pollination to test self-compatibility; (2) intra-morph pollination (illegitimate cross); (3) inter-morph pollination (legitimate cross); (4) netted without hand-pollination; and also (5) marked some flowers without treatments as natural control. At the end of the flowering period, the mesh bags were removed to allow fruits to mature naturally. Three months after pollination, fruit set was recorded and statistical analyses were performed by *G*-test.

Pollen-tube growth was examined *in vivo*. Newly open virgin flowers in the evening were hand-pollinated by fresh pollen with above (1), (2) and (3) treatments. After pollination, the styles with ovary were collected per time interval (1, 3, 6, 12 and 24 h) and fixed in FAA. In lab, after softening in 10% Na_2_SO_3_ (100 °C) for 6 h, pistils stained with aniline blue were observed by fluorescence microscope^75^.

### Pollinators and their host plants

We observed pollinator activities in the Yongxing population during the peak flowering (September to December) of *G*. *speciosa*. Fourteen days’ observations were carried out at 20:00 to 01:00 and 06:30 to 08:30 in three sites of the island. The presence of floral visitors was recorded, and special attention was paid to their visitation behaviors. Visitors which touched the pistil and stamen were recorded as pollinators.

As the host plant is necessary for the moth’s lifecycle, we surveyed the pollinators’ host plants on the Yongxing Island during the same season after confirming the pollinators. We collected the larvae, and fed them in the lab till eclosion to confirm the imago.

### Distribution of *G*. *speciosa*, pollinator and host plant

Ecological Niche Modeling (ENM) was applied to determine the potential geographic distribution of the studied species. ENMs establish relations between the occurrences of species and environmental conditions^76^. Occurrence data for each species were obtained from Global Biodiversity Information Facility (GBIF). We used ENMTools to remove duplicate occurrences based on the resolution of climatic variables, to ensure only one point kept in per grid cell^77^. Nineteen bioclimatic variables for MaxEnt analysis were obtained from the WorldClim website with 2.5 arcmin spatial resolution^78^.

MaxEnt software (v. 3.3.3 K) uses a modeling method called maximum entropy distribution, which estimates the probability distribution for a species’ occurrence based on environmental constraints^79^. Runs were conducted with the default variable responses settings. And a logistic output format results in a map of habitat suitability of the species ranging from 0 to 1, where 0 being the lowest and 1 the highest probability. We selected 75% data for training and the rest 25% for testing. In order to observe and compare the potential distribution of each species, we used the 10th percentile training presence as a suitability threshold^80^, and we assumed that a cell is suitable if its suitability score is greater than the 10th percentile of training presence points. Other values were kept as default. The percentage of overlapping area was calculated by ArcGIS 9.3. The models were evaluated with the area under the curve of a receiver-operating characteristic plot^81^. The current occurrence from GBIF is labeled on the maps to compare the real distribution with the predicted distribution.

## Electronic supplementary material

Supplementary Information
