# Supplementary material for: Pollination niche availability facilitates colonization of Guettarda speciosa with heteromorphic self-incompatibility on oceanic islands
Source: Sci Rep. 2018 Sep 13;8:13765. doi: 10.1038/s41598-018-32143-5 (PMC6137094; doi:10.1038/s41598-018-32143-5)
Supplement: Supplementary file 1 — Supplementary Information [file 41598_2018_32143_MOESM1_ESM.pdf]

Supplementary Information

Pollination niche availability facilitates colonization of *Guettarda speciosa* with heteromorphic self-incompatibility on oceanic islands

Yuanqing Xu[1a] , Zhonglai Luo[1a*], Shaoxiong Gao[2], Dianxiang Zhang[1*]

[1] Key Laboratory of Plant Resources Conservation and Sustainable Utilization, South China Botanical Garden, The Chinese Academy of Sciences, Guangzhou 510650, China

[2] Chongqing Nanshan Botanical Garden, Chongqing 400065, China

[a] These authors contributed equally to this work.

*Correspondence should be addressed to:

Dr. Zhonglai Luo or Prof. Dianxiang Zhang

E-mail: luozhongl @scbg.ac.cn; dx-zhang@scbg. ac.cn

Table S1. The floral characters of *Guettarda speciosa* and Mann-Whitney U-test between L- morph and S-morph.

Flora attribute(mm)	Long-styled morph (N=74)		Short-styled morph (N=42)		Mann-Whitney U-test
	x±SD	Median	x±SD	Median	
Corolla diameter	25.97±3.82	25.4	25.32±4.10	25.8	U=1437，P=0.503
Corolla tube diameter	2.60±0.43	2.6	2.84±0.38	2.8	U=1085.5，P < 0.05
Corolla tube length	30.48±3.42	30.6	39.00±4.96	39.6	U=265.5，P < 0.001
Anther height	27.00±3.56	27.15	36.21±5.26	36.5	U=215.5，P < 0.001
Stigma height	32.45±3.40	32.4	18.77±2.60	18.75	U=6，P < 0.001
Stigma-anther separation	-2.01±1.29	-2.1	15.40±3.69	15	U=0，P < 0.001
Stigma-corolla Separation	1.95±1.77	1.8	-20.22±3.80	-19.5	U=0，P < 0.001
Anther length	3.57±0.46	3.6	3.84±0.49	3.9	U=1063，P < 0.01
Corolla-anther separation	1.76±1.18	1.55	0.45±0.58	0.1	U=443.5，P < 0.001
Stigma diameter	1.48±0.25	1.4	1.16±2.24	1.1	U=496.5，P < 0.001
Stigma length	1.42±0.28	1.4	1.63±0.33	1.75	U=989.5，P=0.001
Polar axis of pollen (μm)	27.46±1.50	27.64	28.24±1.64	28.89	U=296，P < 0.05
Equatorial axis of pollen (μm)	27.31±1.45	27.56	28.61±1.89	28.84	U=343，P < 0.05

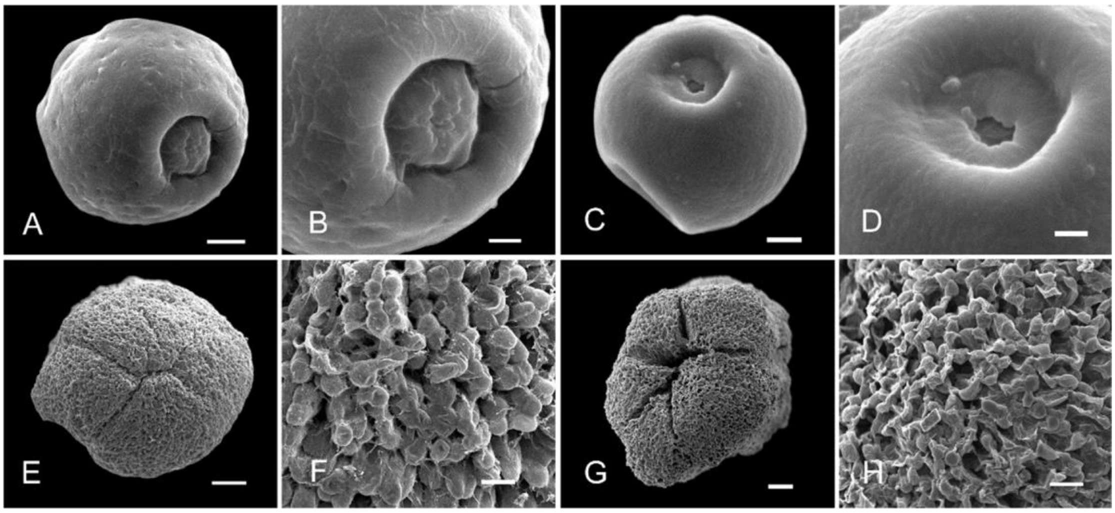

Fig.S1. SEM micrographs of pollen and stigma of *Guettarda speciosa*. A & B: Pollen of L-morph; C & D: Pollen of S-morph; E & F: Stigma of L-morph; G & H: Stigma of S-morph. Bar=5μm in A, C; 2μm in B, D; 250μm in E, G; 25μm in F, H.

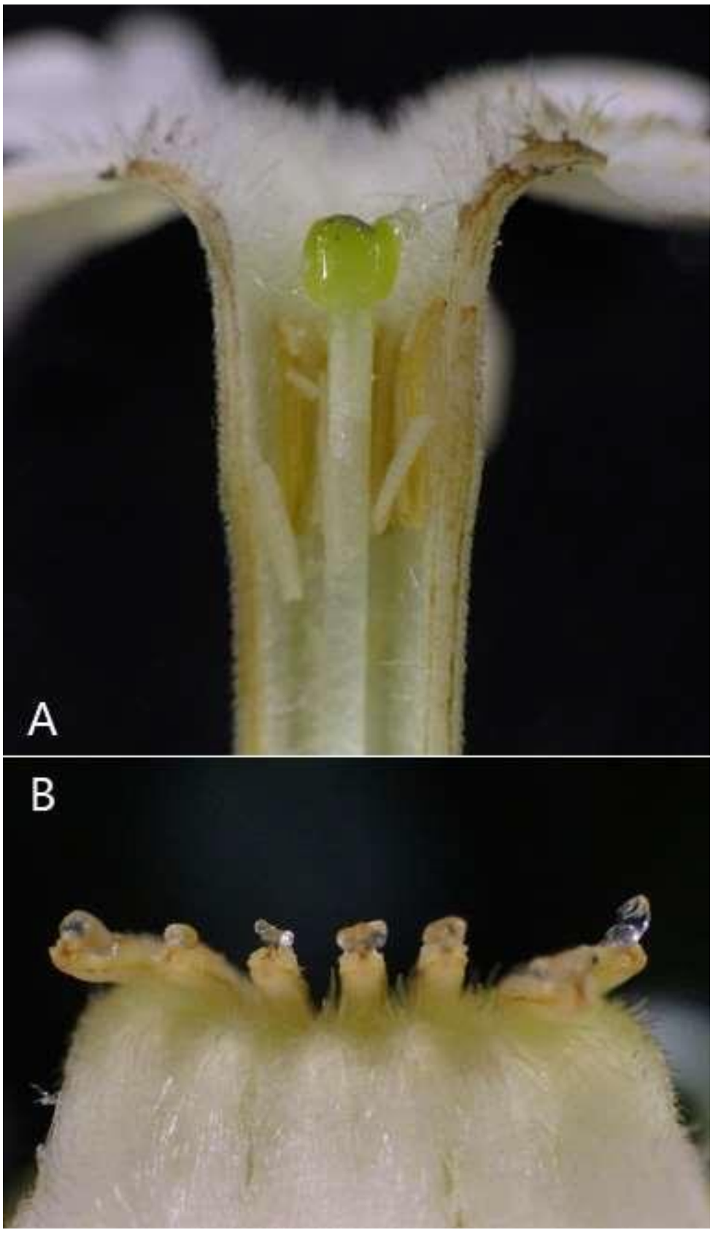

Fig. S2. A: The sticky stigma and pollen clumps; B: Secretion on the anther tips.
